# Genomic Instability: A Stronger Prognostic Marker Than Proliferation for Early Stage Luminal Breast Carcinomas

**DOI:** 10.1371/journal.pone.0076496

**Published:** 2013-10-15

**Authors:** Anne Vincent-Salomon, Vanessa Benhamo, Eléonore Gravier, Guillem Rigaill, Nadège Gruel, Stéphane Robin, Yann de Rycke, Odette Mariani, Gaëlle Pierron, David Gentien, Fabien Reyal, Paul Cottu, Alain Fourquet, Roman Rouzier, Xavier Sastre-Garau, Olivier Delattre

**Affiliations:** 1 Department of Tumor Biology, Institut Curie, Paris, France; 2 INSERM U830, Institut Curie, Paris, France; 3 Department of Translational Research, Institut Curie, Paris, France; 4 Department of Biostatistics, Institut Curie, Paris, France; 5 INSERMU900, Institut Curie, Paris, France; 6 Ecole des Mines de Paris, Paris, France; 7 AgroParisTech, UMR518, Paris, France; 8 Department of Surgery, Institut Curie, Paris, France; 9 Department of Medical Oncology, Institut Curie, Paris, France; 10 Department of Radiation Oncology, Institut Curie, Paris, France; University of North Carolina School of Medicine, United States of America

## Abstract

**Background:**

The accurate prognosis definition to tailor treatment for early luminal invasive breast carcinoma patients remains challenging.

**Materials and Methods:**

Two hundred fourteen early luminal breast carcinomas were genotyped with single nucleotide polymorphisms (SNPs) array to determine the number of chromosomal breakpoints as a marker of genomic instability. Proliferation was assessed by KI67 (immunohistochemistry) and genomic grade index (transcriptomic analysis). IHC3 (IHC4 score for HER2 negative tumors) was also determined.

**Results:**

In the training set (109 cases), the optimal cut-off was 34 breakpoints with a specificity of 0.94 and a sensitivity of 0.57 (Area under the curve (AUC): 0.81[0.71; 0.91]). In the validation set (105 cases), the outcome of patients with > 34 breakpoints (11 events / 22 patients) was poorer (logrank test *p* < 0.001; Relative Risk (RR): 3.7 [1.73; 7.92]), than that of patients with < 34 breakpoints (19 events / 83 patients).Whereas genomic grade and KI67 had a significant prognostic value in univariate analysis in contrast to IHC3 that failed to have a statistical significant prognostic value in this series, the number of breakpoints remained the only significant parameter predictive of outcome (RR: 3.47, Confidence Interval (CI [1.29; 9.31], *p* = 0.014)) in multivariate analysis .

**Conclusion:**

Genomic instability, defined herein as a high number of chromosomal breakpoints, in early stage luminal breast carcinoma is a stronger prognostic marker than proliferation.

## Introduction

A major challenge of breast cancer treatment is to accurately identify patients in whom adjuvant therapy can be safely avoided [1]. Over the past decade, five molecular groups of invasive breast carcinoma have been identified, each associated with a different outcome [2]. Triple-negative carcinomas are recognized as the group associated with the poorest prognosis and efficiently treated with adjuvant chemotherapy [3,4]. Identification of high-risk patients in this group by means of the classical clinicopathological parameters therefore remains difficult. As a result of mammography screening, the majority of breast cancers are now diagnosed at an early luminal stage with no axillary lymph node metastasis, and HER2-negative. The benefit of medical adjuvant treatment in these patients also remains controversial [1]. While luminal carcinoma patients display various outcomes according to their tumor proliferation rate and clinical stage, they harbor a constellation of driver gene mutations and chromosomal alterations [5–9].

Proliferation assessed either by mitotic index according to the Ellis and Elston recommendations [10] or KI67 immunostaining improves the prognostic definition of luminal breast carcinoma patients [11]. However, molecular methods such as genomic grade index (Mapquant®)[12] have been proposed as a more accurate tool to evaluate proliferation. This technique has been developed to classify grade 2 tumors into good and poor outcome groups. IHC4 score, an algorithm inferred from ER, PR, KI67 and HER2 immunostainings has been recently proposed as an interesting alternative tool to refine breast cancers prognostic. Its prognostic value was found to be equivalent to that of the OncotypeDX® for a lower cost [13]. 

Pangenomic analyses have provided substantial data describing breast carcinoma patterns of genomic alteration [9,14,15]. These analyses, consolidated by recent exome-sequencing results, have also shown that genomic alterations are different according to the molecular or histological types of breast carcinomas [5–7]. 

The prognostic value of genomic instability for breast cancer has been evaluated either by transcriptomic signature enriched with genes involved in chromosomal integrity maintenance [16–19] or inferred evaluation of ploidy based on fluorescent in situ hybridization [20]. 

We and others have proposed that the level of genomic complexity within breast carcinomas could contribute to define patient outcome [16,20–23]. In our previous work, we determined that the number of breakpoints had a prognostic power to identify high-risk T1-T2 N0 invasive ductal carcinomas of the breast. We used an in-house array-comparative genomic hybridization (CGH) tool to define a CGH classifier. This classifier was based both on the number of chromosomal breakpoints together with three regions (2p22.2, 3p23 and 8q21-24) to discriminate tumors associated with good and poor outcome. The sensitivity of this classifier was 66%, with a specificity of 84% and an accuracy of 78% [21]. 

The objective of the present study was to validate, in an independent series, the prognostic value of our previously established genomic signature. In this study, we used a different tool, the SNPs array (SNP 6.0 array, Affymetrix®), as this array has a higher resolution than the array CGH used in our previous study [21] and compared it to proliferation prognostic value. We focused our analysis on early luminal HER2-negative node-negative breast cancers, i.e. in the population of patients in whom prognosis assessment remains challenging, in whom treatment must be adapted to tumor biology and decreased as far as possible to minimize the side effects of chemotherapy.

## Materials and Methods

### Patients and tumors

All experiments were performed retrospectively and in accordance with the French Bioethics Law 2004-800, the French National Institute of Cancer (INCa) Ethics Charter and after approval by the Institut Curie review board and ethics committee (Comité de Pilotage of the Groupe Sein). In the French legal context, our institutional review board waived the need for written informed consent from the participants. Moreover, women were informed of the research use of their tissues and did not declare any opposition for such researches. Data were analyzed anonymously.

This analysis was confined to ER-positive, node-negative early (T1-T2 tumor smaller than 30 mm), HER2-negative breast cancers because they now represent the majority of breast carcinomas at diagnosis as a result of systematic screening procedures in western countries [24]. Tumours from the two sets (training and validation) were selected among the 7469 tumor samples from patients treated between 1989 and 1999 with a conservative surgery followed by radiation therapy at the Institut Curie for T1T2 breast carcinoma. The selection criteria for the two sets were small size (

**Figure 1:**
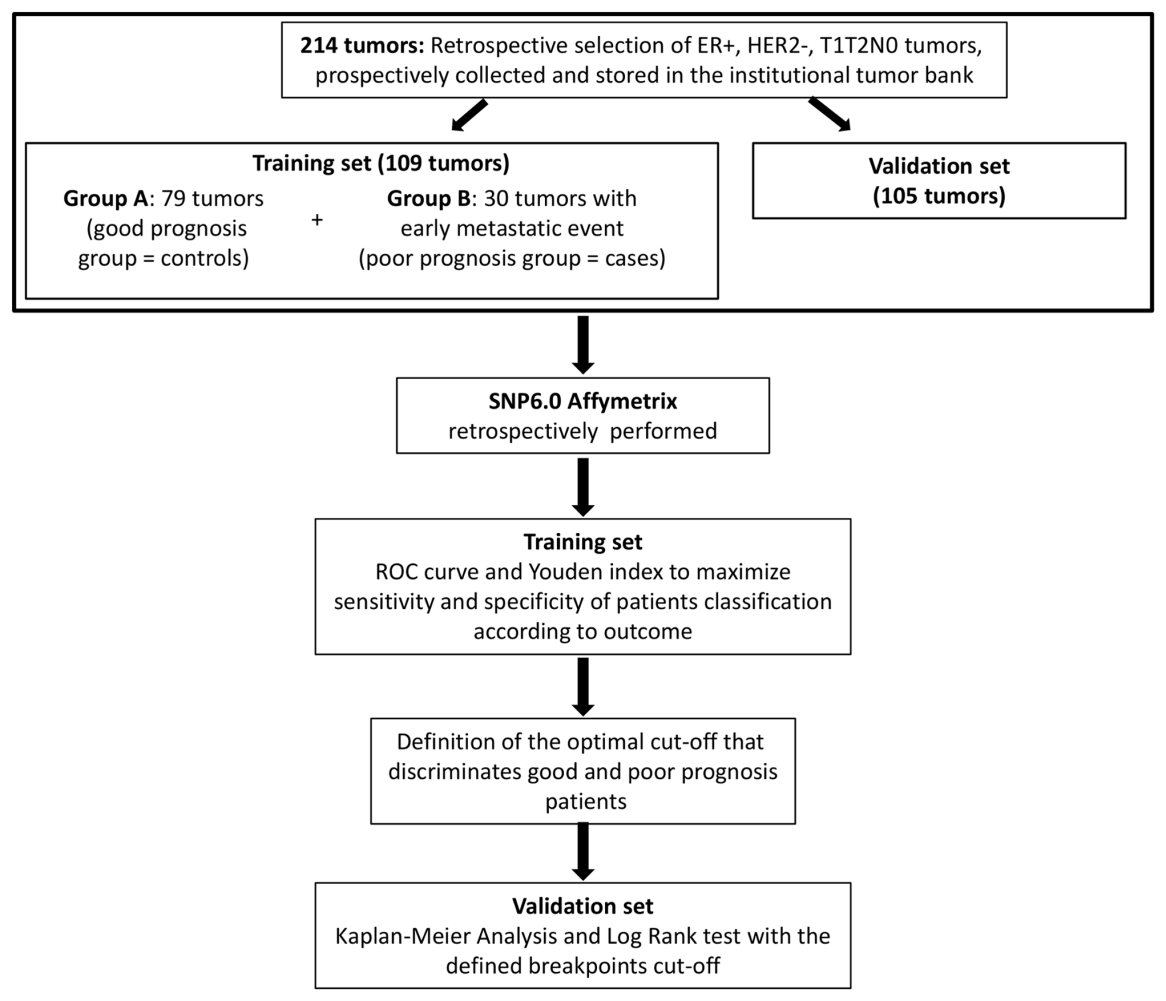
Flowshart of patients and tumor’s selection and design of the study.

< 3cm), node-negative, invasive ductal breast carcinomas ER + HER2-, node negative. Patients older than 75 years, with previous personal history of cancer, with familial history of breast cancer, with multifocal or bilateral tumours, or with initial metastatic disease were excluded from the study. The final selection was based on the availability and quality of tumor samples in our tumor bank. These samples were collected from fresh surgical specimens by a pathologist within one hour after surgery, flash-frozen in liquid nitrogen and stored at -80°C. The Figure 1 represented the workflow for retrospective tumor selection among available frozen samples collected prospectively between 1989 and 1999. Training set consisted of a case-control group composed of 109 tumors. The median follow-up for the patients of this good prognosis group was 10.9 years, (95% CI: 10.4-11.5). Seventy-nine patients were metastasis-free at five years (A good prognosis group, controls). Thirty patients developed metastasis before four years (Bpoor prognosis group, cases) with a median time to metastasis of 2.3 years (interquartile range: 1.5-3.4). None of the patients had received neoadjuvant or a

djuvant chemotherapy, but six patients had received adjuvant hormone therapy. 

The validation set was composed of 105 tumors samples from 105 patients. Median follow-up was 10.5 years, (95% CI: 10.1-10.9). Tumors were invasive ductal carcinomas smaller than 30 mm, ER-positive, HER2-negative, and node-negative. None of the 105 patients had received neoadjuvant or adjuvant chemotherapy and ten patients had received adjuvant hormone therapy. Thirty out of the 105 patients developed loco-regional or contralateral relapses or experienced a metastatic event (8 out of the 30 patients).

All tumors were retrospectively reviewed by pathologists experienced in breast pathology (XSG and AVS) for the purpose of this study.In particular, histoprognostic grade was assessed retrospectively according to the recommendations of Elston and Ellis [25].

### Immunostaining for Estrogen-receptor (ER), progesterone-receptor (PR), HER2 and KI67 determination

ER, progesterone receptor, HER2 receptor and KI67 status were assessed by immunohistochemistry on representative formalin-fixed tumor blocks, according to previously published protocols[26] (see Data S1 for antibody details). The semiquantitative KI67 assessment was performed as previously published [27] and as recommended [28]. A cut-off of 14% was used to define tumors with a high KI67 score (according to St Gallen recommendations [29] and cut-off for molecular classification[30]. Internal (normal glands surrounding the carcinoma) and external controls (for ER, PR and HER2: tissue-microarrays composed of tumors with known ER, PR status, and known numbers of *HER2* gene copies together with normal mammary tissue; for KI67: normal lymph node with germinal centers as positive controls) were included in all immunostaining experiments. 

Following Cuzick’s recommandations, the IHC4 was determined and adapted for HER2 negative tumors (named “IHC3”) and, as KI67 was assessed manually, in the formulae, the KI67 value was rescaled [13]. 

### DNA and RNA analyses

All tumor samples contained more than 50% of cancer cells, as assessed by Hematein & Eosin staining of histologic sections of the frozen samples used for nucleic acid extraction. DNA and RNA were extracted according to a previously described phenol-chloroform protocol [27,31]. 

### SNP6.0 analyses for tumor genomic complexity evaluation

The Affymetrix GeneChip Human Mapping 6.0^®^ was normalized using Affymetrix Genotyping Console^®^ (version GTC 3.0.1) (.cel files and data available under accession number GSE48064 in GEO database).The signal from SNP probes was then segmented using Colibri [32]. A first segmentation round was performed to identify and discard outlier probes giving rise to singleton segments. The number of breakpoints was estimated according to the Zhang and Siegmund’s BIC-based model selection criterion (Data S2) [33]. 

The optimal segmentation in terms of least-square fit was recovered via dynamic programming. The pruned version proposed in [[32]] can be used to achieve this optimization step with almost linear complexity. We used the implementation available in the cghseg package [34] via the function segmeanCO. The training set was used to assess the link between the number of breakpoints and the tumor group (A: late onset versus B: early onset metastatic or relapse event). The breakpoint number (assessing the number of chromosomal segmental alterations) cut-off able to accurately identify B tumors (poor prognosis tumors) was determined using the Youden index. This index is defined as the threshold that maximizes the sum of sensitivity and specificity. The number of breakpoints was used as a marker of genomic complexity.

### Expression analyses and determination of genomic grade index

After RNA quality control and according to a previously published protocol [27], 94 of the 105 validation cases meeting our selection criteria for node status, tumor size, ER and HER2 status, were hybridized onto U133 plus 2.0 Affymetrix® chips. Transcriptomic data were normalized using RMA (under R software 2.13.2 version). The genomic grade index (GGI), a continuous variable, was calculated using the MapQuant DxM protocol, according to Sotiriou et al [12] and as previously described [27], and was defined as GGI = scale [sum (Probe Sets up in Grade 3 tumors-112 probe sets)- sum (Probe Sets up in Grade 1 tumors-16 probe sets)- offset]. Scale and offset are transformation parameters to standardize the genomic grade index values. The MapQuant® GGI was then standardized by setting the scale and offset parameters so that the mean GGI of histologic Grade 1 tumors was -1 and that of histologic grade 3 tumors was +1. The cut-off was set at 0. Based on the value of the GGI, a genomic grade (Genomic Grade 1 or Genomic Grade 3) was then attributed to each tumor sample.

## Results

### Patients

Detailed patient and tumor characteristics are displayed in Table 1. Training and validation sets comprised a large majority of T1, grade 1 and 2 tumors. 

**Table 1:** Clinico-biological characteristics of patients and tumors.

**Characteristics**	**Training set (n=109)**	**Validation set (n=105)**
**Age (years)**		
Means (min-max)	55.6 (36-75)	54.2 (40-69)
>50	74 (68%, 95%CI : 59%-77%)	68 (65%, 95%CI : 56%-74%)
<50	35 (32%, 95%CI : 23%-41%)	37 (35%, 95%CI : 26%-44%)
**Size**		
T1	77 (71%, 95%CI :62%-79%)	77 (73%, 95%CI : 65%-82%)
T2	32 (29%, 95%CI : 20%-38%)	28 (27%, 95%CI : 19%-35%)
**Elston-Ellis grade**		
I	63 (58%, 95% CI:49%-67%)	48 (46%, 95% CI : 36%-55%)
II	35 (32%, 95%CI:23%-41%)	41 (39%, 95%CI : 30%-48%)
III	9 (8%, 95%CI :3%-13%)	16 (15, 95%CI : 8%-22%)
Missing values	2 (2%)	0
**Vascular invasion**		
Yes	10 (9%, 95%CI:4%-14%)	20 (19%, 95%CI :12%-26%))
**ER status (IHC)**		
Positive	109 (100%)	105 (100%)
**PR status (IHC)**		
Positive	83 (76%, 95%CI:68%-84%)	80 (76%, 95%CI:68%-84%)
**KI67**		
< 14%	nd	47 (45%, 95%CI:36%-55%)
> 14%	nd	58 (55%, 95%CI:46%-64%)
**Genomic grade**		
GGI 1	nd	56 (60%, 95%CI:50%-69%)
GGI 3	nd	38 (40%, 95%CI:30%-49%)
Undetermined	nd	11 (10.5%)

Min: minimum; max: maximum. GG: genomic grade; **ER**: **estrogen** **receptors**; **PR**: **progesterone** **receptor**; **95%CI**: **Confidence** **Interval.**

In addition to the Elston and Ellis histoprognostic grade, proliferation was also evaluated by KI67, IHC3 and genomic grade index for 94 of the 105 tumors in the validation set. As shown in Table 1, 45% and 60% of these tumors presented a KI67 index less than 14% of positive cells and a genomic grade index of 1, respectively.

### Assessment of genomic complexity with the number of chromosomal breakpoints and definition of a classifier within the training set

**Figure 2:**
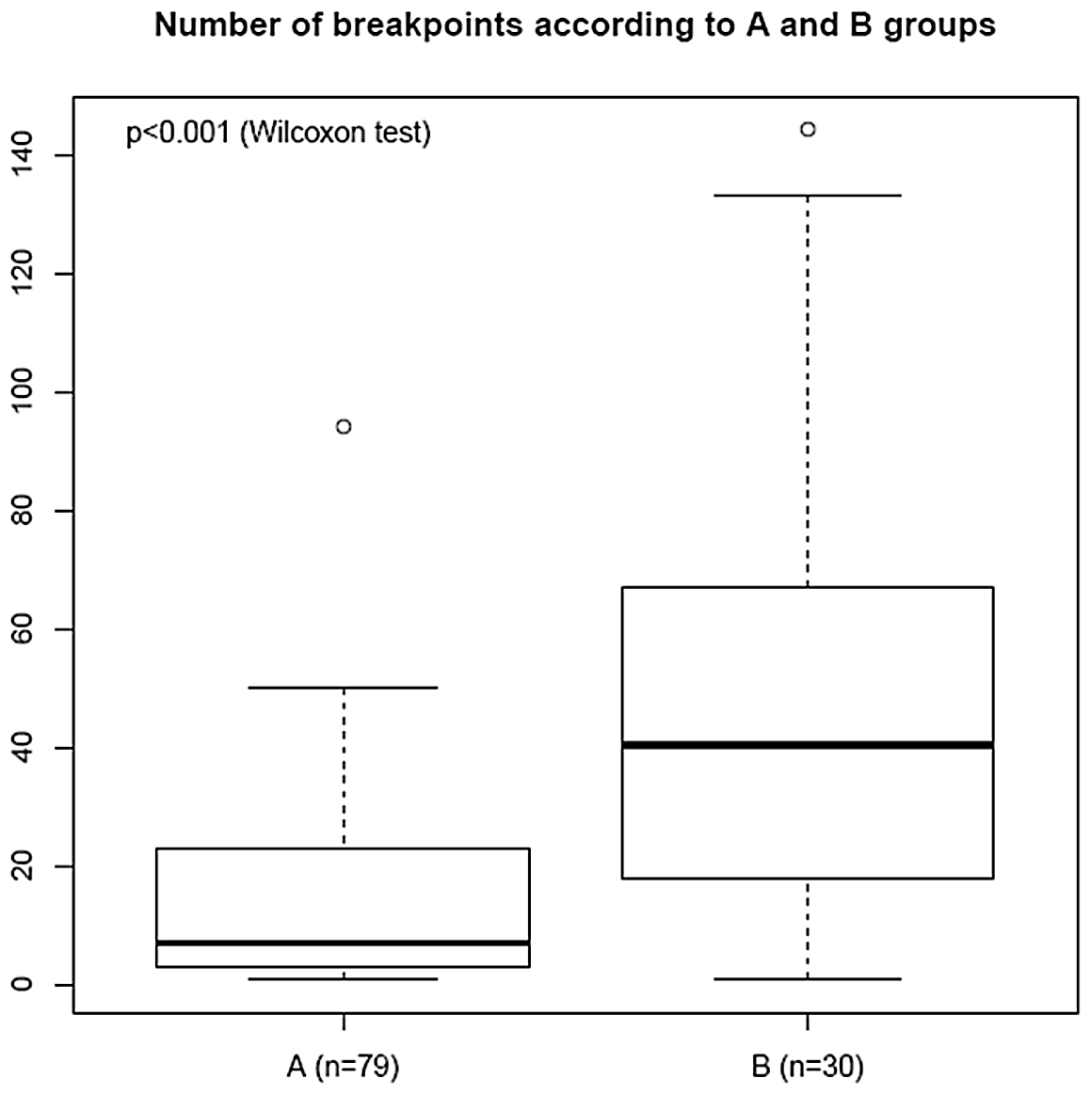
Boxplot of the number of breakpoints within groups A and B from the training set. The black line represents the breakpoints median values (median = 7 in A group; median = 40.5 in B group). The bottom and top of the boxes represent the 25th and 75th percentile respectively, whereas the box represents the interquartile range. The whiskers extend to the most extreme data point which is no more than 1.5 times the interquartile range from the box. The single point at the top of each boxplot represents the maximum number of breakpoints within each group A and B respectively.

According to the segmentation protocol described above, the numbers of breakpoints was determined in group A and group B tumors of the training set and were compared. As shown in Figure 2, the median number of breakpoints in group A tumors was significantly lower (median value=7) than the median number of breakpoints in group B (median value = 40.5)(95% CI: -39.00 ;-14.00, *p<0.001*, Wilcoxon test).

An optimal cut-off of thirty-four breakpoints was defined to assign each patient to a risk class, as the number of breakpoints ensuring maximum sum of sensitivity (0.57 95% CI: 39%-74%) and specificity (0.94 95% CI: 88%-99%), was 34. A tumor with more than 34 breakpoints was classified in the high-risk complexity group, otherwise it was assigned to the low-risk complexity group. In good prognosis group (no metastatic event within five years), 74 tumors (74/79, 94% 95% CI: 88%-99%) were considered to be low-risk complexity tumors and 5 were considered to be high-risk complexity tumors (5/79, 6% 95% CI: 0%-12%) whereas in poor-prognosis group (metastatic relapse in less than four years), 13 tumors were considered to be low-risk complexity tumors (13/30, 43% 95% CI: 26%-61%) and 17 tumors (17/30, 57% 95% CI: 39%-74%) were considered to be high-risk complexity tumors (Table 2).

**Table 2:** Number of tumors with less or more than 34 breakpoints (Threshold) within A and B groups, specificity and sensibility of tumor classification in the training set.

**Groups**	**Number of breakpoints**		**Specificity**	**Sensitivity**
	**< 34 (%**)	**> 34 (%**)		
A	74 (94)	5 (6)	0.94	
B	13 (43)	17 (57)		0.57

**Figure 3:**
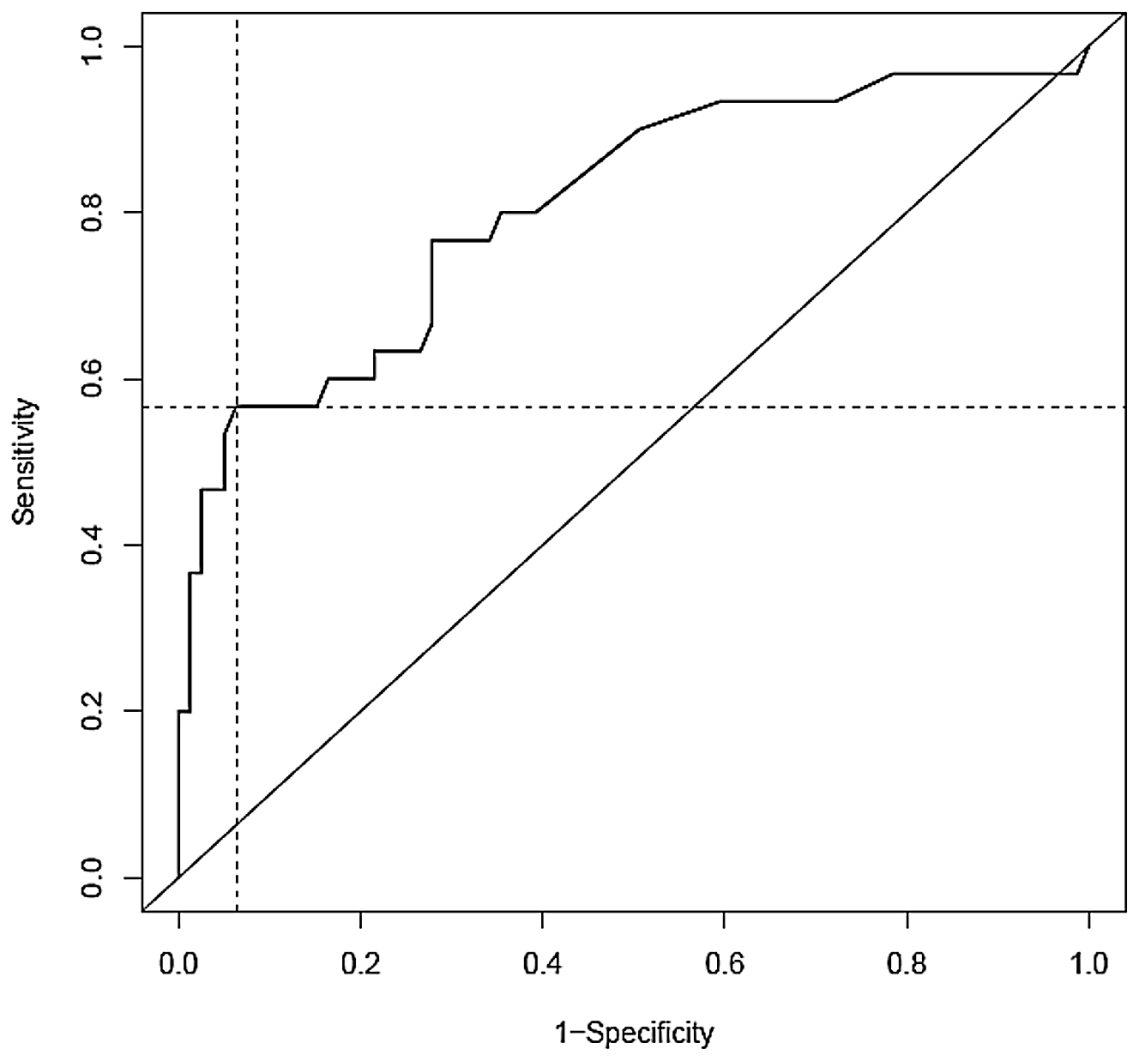
Receiving Operating Curve (ROC) curve for the number of breakpoints threshold determination. Intersection of dotted-lines = Youden index.

Analysis of the complexity classifier produced a Receiving Operating Characteristics (ROC) curve with a significant area of 0.81 (95% CI: 0.71-0.91) (Figure 3). 

### Number of breakpoints is linked with histoprognostic grade, genomic grade and KI67 labeling

In the validation set of this series of cases, a link between grade (p<0.001; Kruskal-Wallis test), genomic grade (p<0.001, Wilcoxon test), KI67 (p<0.001, Wilcoxon test) and the number of breakpoints was observed. 

The median number of breakpoints in the group of tumors with a KI67 smaller than 14% and greater than or equal to 14% were 5 and 17, respectively, (95% CI:-19-4) (Figure S1). 

The Spearman correlation coefficient between genomic grade index and number of breakpoints was 0.54 (*p* < 0.001) (Figure S2).

### Link between the number of breakpoints according to the cut-off chosen and disease-free interval in the validation set

To test the robustness of this cut-off, we determined disease-free interval in the validation set and tested the correlation with the number of breakpoints in univariate and multivariate analyses taking into account clinicopathological and proliferation markers such as grade, KI67 GGI and IHC3 (Table 3). 

**Table 3:** Univariate and multivariate analyses for disease free-interval in the validation set.

Variables	Univariate analysis		Multivariate analysis	
	Relative Risk (95% CI)	*p*	Relative Risk (95% CI)	*p*
Age <=50 vs >50 years	0.94 (0.45-1.98)	*0.873*		
Pathological size >20mm vs <=20mm	1.11 (0.42-2.92)	*0.827*		
Grade II/III vs I	2.12 (0.99-4.5)	*0.052*	1.39 (0.52-3.70)	*0.515*
PR status Negative vs Positive	0.9 (0.38-2.1)	*0.805*		
Vascular invasion Yes vs No	1.36 (0.55-3.36)	*0.512*		
KI67(IHC) >14 vs < 14%	2.57 (1.16-5.68)	*0.020*	1.39 (0.48-4.08)	*0.544*
Genomic Grade GG3 vs GG1	2.59 (1,17-5.73)	*0.019*	1.16 (0.39-3.48)	*0.788*
IHC3 score (continuous)	1.01 (1.00-1.01)	*0.204*		
Number of Breakpoints >34 vs <34	3.7 (1.73-7.92)	*<0.001*	3.47 (1.29-9.31)	*0.014*

vs: versus.

**Figure 4:**
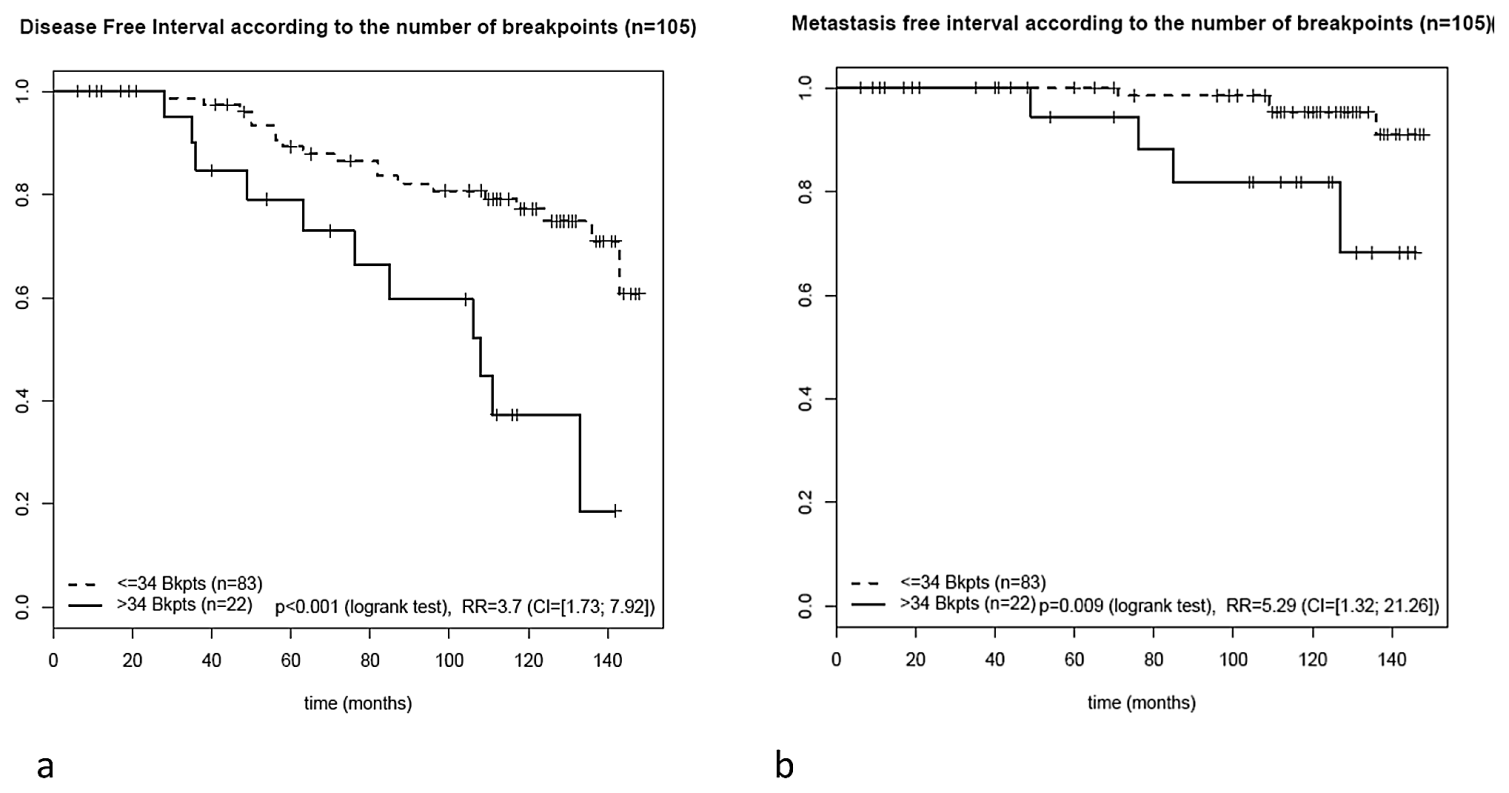
Disease free-interval (a) and metastasis free-interval (b) analyses (Kaplan Meier analysis and log rank test) in the validation set.

Whereas IHC3 score was not found statistically significant in this series of tumors, the three proliferation markers, histopronostic grade, KI67 and genomic grade index together with the number of breakpoints were significantly associated with disease free interval at a significance level of 10% in univariate Cox analysis (Table 3). Interestingly, the number of breakpoints remained the only significant parameter when tested with grade, KI67 and genomic grade index in multivariate Cox analysis (relative risk of 3.47, 95% CI:1.29–9.31, p= 0.014, Wald test). Patients with more than 34 breakpoints experienced a statistically shorter disease-free interval (Log rank test *p<0.001*), as determined by the Cox model and Kaplan-Meier analyses (Figure 4a). 

The number of breakpoints was significantly predictive of metastasis-free interval 

(

< 34 breakpoints: 4 events in 83 patients; > 34 breakpoints: 4 events in 22 patients (*p=0.009*, logrank test; RR: 5.29 [1.32; 21.26]) (Figure 4b).

## Discussion

In this study, we assessed and validated the prognostic power of a genomic complexity signature based on the number of breakpoints determined with a pan-genomic SNP array in early stage node-negative luminal HER2-negative invasive ductal breast cancers. The number of breakpoints in this series outperformed the prognostic value of histoprognostic grade, KI67 index and genomic grade of the tumors.

Using high resolution genome-wide tools from different platforms, Russnes and colleagues [22] showed that, within the different molecular subtypes, qualitative genomic alterations and complex arm aberration index identified tumors associated with breast cancer-specific death. Others groups have shown the prognostic value of chromosomal instability score evaluated as the number of gains and losses assessed on SNP arrays, either in ER positive or negative tumors [16,20,23]. However, these analyses did not focus on early stage (T1 and small T2 N0) luminal tumors. 

In our previous work, we have identified a DNA-based genomic signature [21] on T1-T2 breast carcinomas but that encompassed ER and HER2 positive and negative cases. In the present study focused specifically on early stage luminal breast carcinomas, we have further demonstrated the prognostic value of the number of chromosomal breakpoints to assess chromosomal instability using a SNP-based array. ER-positive and HER2-negative, node-negative early stage breast carcinomas are the commonest form of breast cancers in the context of systematic mammography screening. Tailoring treatment for these patients is mandatory but challenging [35]. The challenge is to identify poor prognosis patients when classical anatomical parameters such as size and node status are irrelevant and when the tumors are luminal [36]. The specificity of this new DNA-based signature was increased from 84% to 94%, which means that at initial diagnosis, this signature can contribute to accurate identification of good prognosis breast cancers, i.e. those with 34 breakpoints or less. This signature takes into account the number of breakpoints and not regions of gains or losses. Indeed, luminal invasive carcinomas not otherwise specified demonstrate highly heterogeneous genomic profiles [5] with numerous chromosomal gains and losses, but which are never observed in all cases in the same combination [14,22,34]. We therefore preferred to develop a quantitative signature than a qualitative assessment of the alterations. This approach is more easily reproducible using a standardized bioinformatic tool that can be applied in a customized fashion and is readily accessible [34].

Other authors and we have proposed the use of proliferation as a powerful prognostic tool to identify those tumors with poor outcome [27,28,36]. However, the present study showed that the number of chromosomal breakpoints in multivariate analysis remained the only statistically significant parameter to identify poor prognosis early invasive luminal HER2-negative and node-negative breast cancers, and was more reliable than proliferation assessed by either grade, KI67, IHC3or genomic grade.

Signature such as Oncotype DX® [37] has also been proposed as a robust tool to classify luminal breast carcinomas into three different prognostic groups. However, although the proliferation set of genes is robust and based on five genes encompassing KI67, the HER2 set of genes is more controversial [38], it is of no value in HER2-negative carcinomas. In addition, this test dramatically increases the cost of the diagnostic procedure [39] and it adds little prognostic information compared to the recently proposed cheaper IHC4 test [13,40,41]. 

In that context, we propose that determination of the number of breakpoints should help to refine the prognosis of early stage breast tumors [21,22]. Even though, the DNA-based signature is correlated with proliferation, it also assesses a different level of biological alterations and provides the global genomic profile of the tumor in a single experiment. It therefore allows an identification of the regions of amplifications, some of which encompass drivers genes that encode for druggable targets [42]. Furthermore, recent developments of Affymetrix® arrays performed with DNA extracted from paraffin-embedded tissue provide promising and robust results like it has been obtained with other arrays [43]. 

## Conclusion

 This study demonstrates that genomic complexity assessed by SNPs arrays and determined as the number of breakpoints can be used to predict the outcome of early stage luminal HER2-negative invasive breast carcinomas. But the use of chromosomal breakpoints number in clinical practice will require a prospective validation of its prognostic value in larger and multicentric series of breast cancer patients. 

## Supporting Information

Data S1 **Antibodies used for tumor characterization.**
(DOCX)

Data S2 **Mathematical analysis for SNP6.0 data segmentation.**
(DOCX)

Figure S1 **Number of breakpoints according to KI67.**
(PDF)

Figure S2 **Gene expression grade according to number of breakpoints.**
(PDF)
