# Supplementary material for: Genomic Instability: A Stronger Prognostic Marker Than Proliferation for Early Stage Luminal Breast Carcinomas
Source: PLoS One. 2013 Oct 15;8(10):e76496. doi: 10.1371/journal.pone.0076496 (PMC3797106; doi:10.1371/journal.pone.0076496)
Supplement: Data S1 — Antibodies used for tumor characterization. (DOCX) [file pone.0076496.s001.docx]

**Supplementary data 1: Antibodies used for tumor characterization:**

- ER, clone 6F11, 1/200, Novocastra, Menarini, Rungis, France, cut-off: > 10% of positive cells
- PR, clone 1A6, 1/200, Novocastra, Menarini, Rungis, France, cut-off: > 10% of positive cells
- HER2 (clone CB11, 1/1000, Novocastra, Menarini, Rungis, France, cut-off: score 0/1+: less than 10% of positive cells with weak staining {Wolff, 2007 #2441})
- Clone MIB1, 1/100, Dako A/S, Glostrup, Denmark
