# Supplementary material for: Genomic Instability: A Stronger Prognostic Marker Than Proliferation for Early Stage Luminal Breast Carcinomas
Source: PLoS One. 2013 Oct 15;8(10):e76496. doi: 10.1371/journal.pone.0076496 (PMC3797106; doi:10.1371/journal.pone.0076496)

**Supplementary data 2: Mathematical analysis for SNP6.0 data segmentation.**

We used for segmentation of SNP6.0 data the methods proposed by Zhang and Siegmund (2007, Biometrics). They discussed that in the context of change-point problems, the traditional BIC does not satisfy the technical assumptions of Schwarz (1978, Annals of Statistics). They proposed a modification to improve its performance.

Consider a sequence of observations y=(y_1_,y_2_,…y_T_) where y_i_ are independently distributed Gaussian random variables:

$y_{i} \text{\textasciitilde}N\left(\mu_{j},\sigma^{2}\right)$ for *i* = *τ_j_* + 1,… *, τ_j_*_+1_*, j*= 0,…,m

Here we will refer to the *τ* ’s as the breakpoints of the DNA copy number profile. m is the number of breakpoints of the DNA copy number profile. T is the number of SNPs of the profile. Of course the change-points are constrained to lie in the set


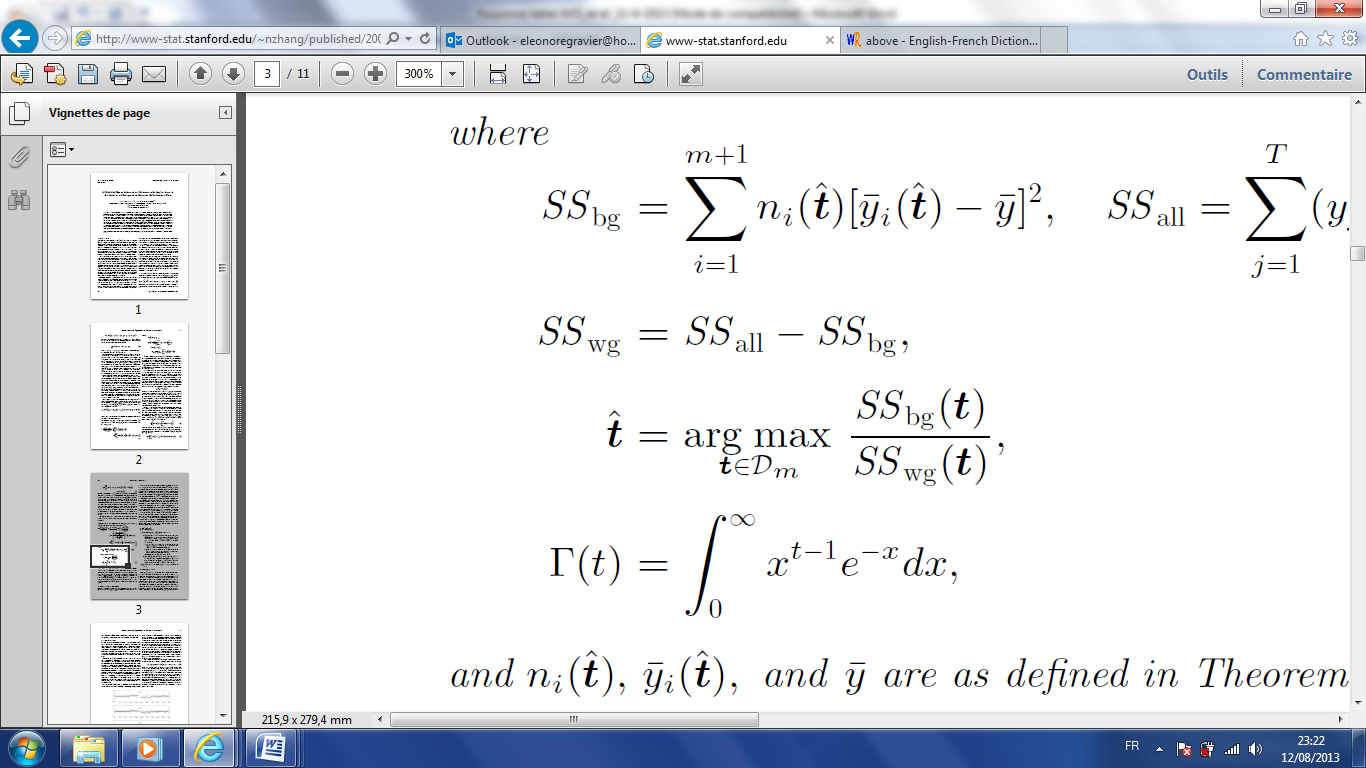
=*{*(*t*_1_*, . . . , t_m_*) : 0 *≤ t*_1_ *≤ t*_2_ *≤ · · · ≤ t_m_ ≤ T}.*


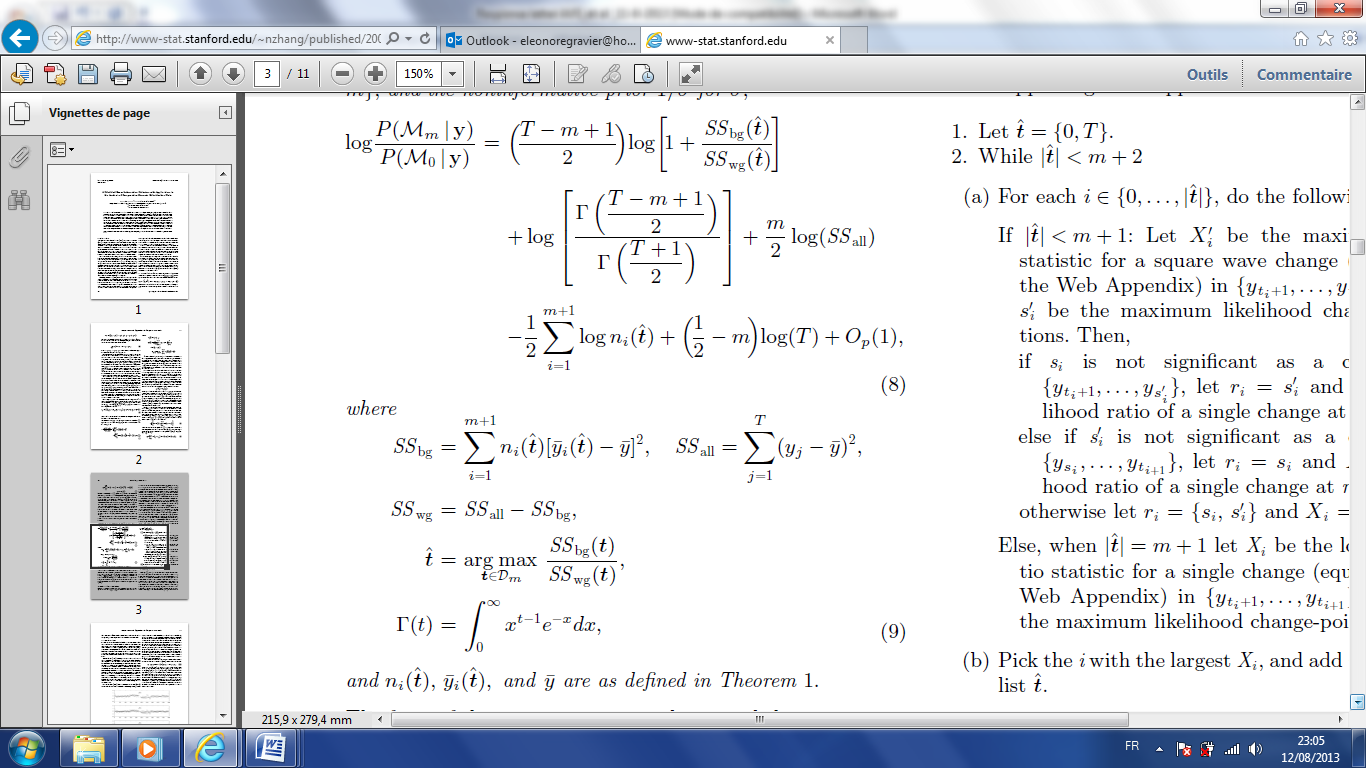
 is the Gaussian model described above with *m* breakpoints.
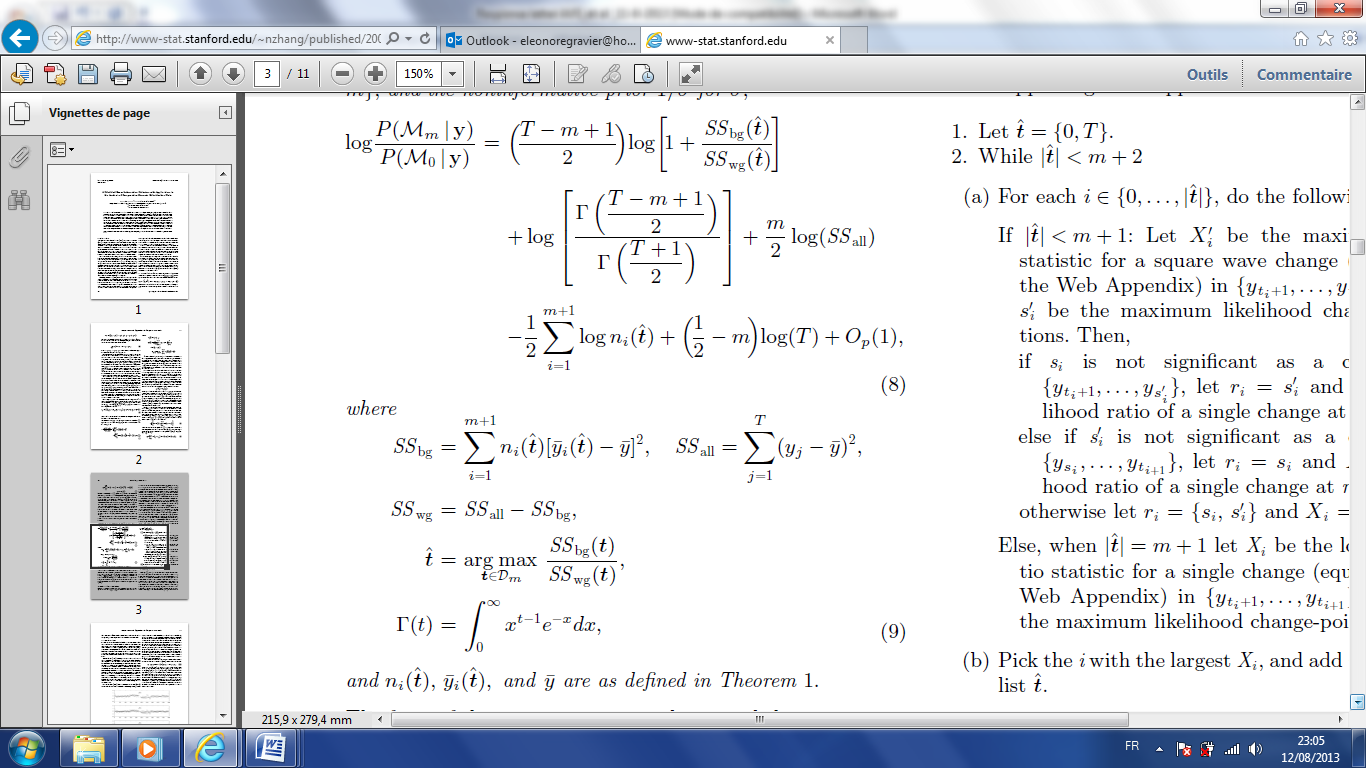
 is the simplest model with no breakpoint.

The modified BIC is derived as an asymptotic approximation of the Bayes factor and is of the form:


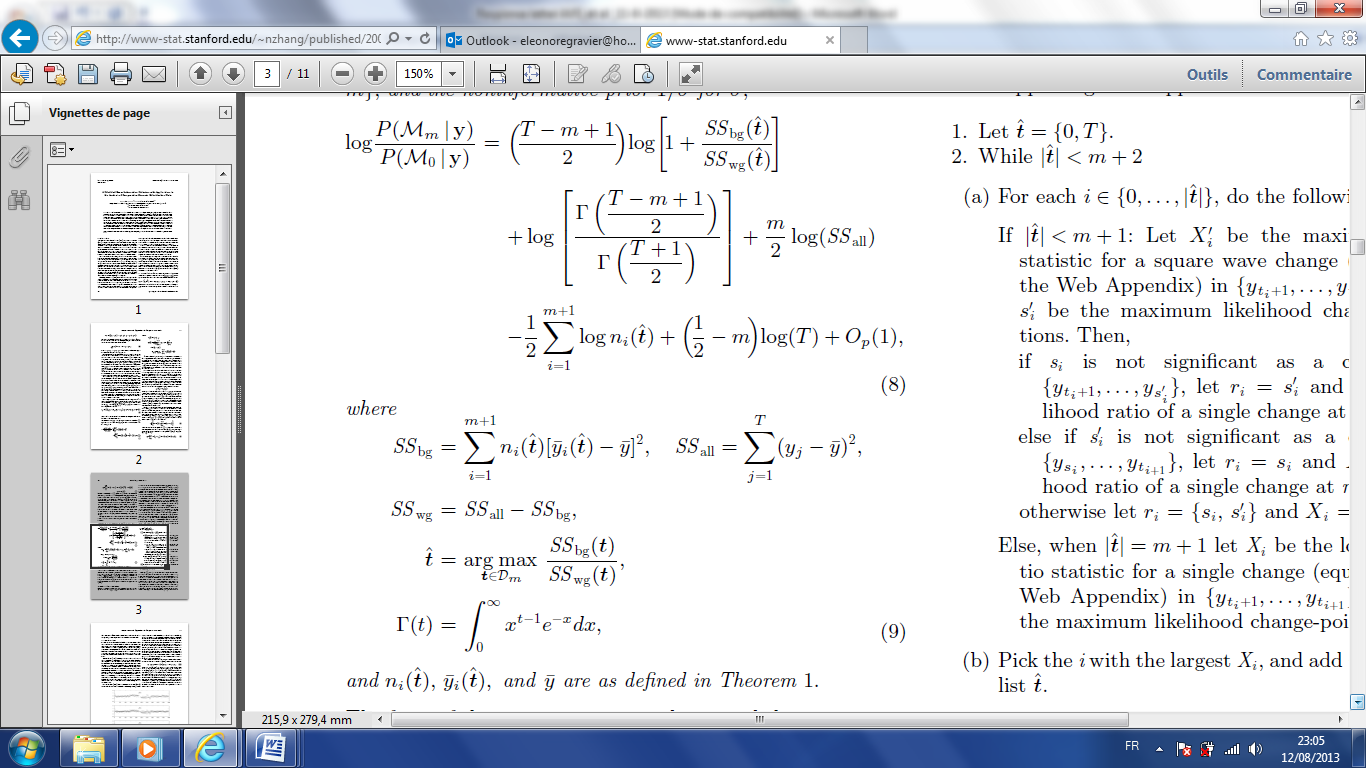


and


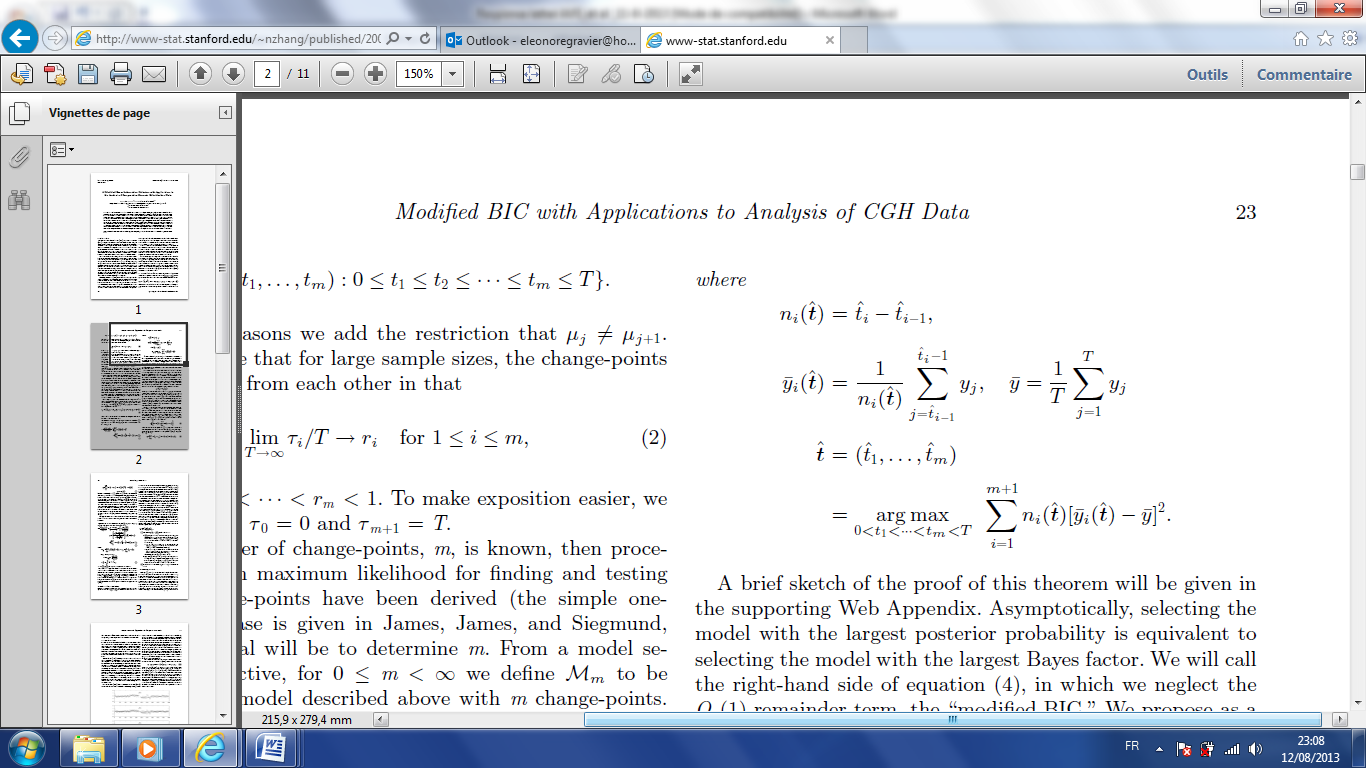

Supplement: Data S2 — Mathematical analysis for SNP6.0 data segmentation. (DOCX) [file pone.0076496.s002.docx]
