# Supplementary figures and images for: Genomic Instability: A Stronger Prognostic Marker Than Proliferation for Early Stage Luminal Breast Carcinomas
Source: PLoS One. 2013 Oct 15;8(10):e76496. doi: 10.1371/journal.pone.0076496 (PMC3797106; doi:10.1371/journal.pone.0076496)

## Number of breakpoints according to ki67

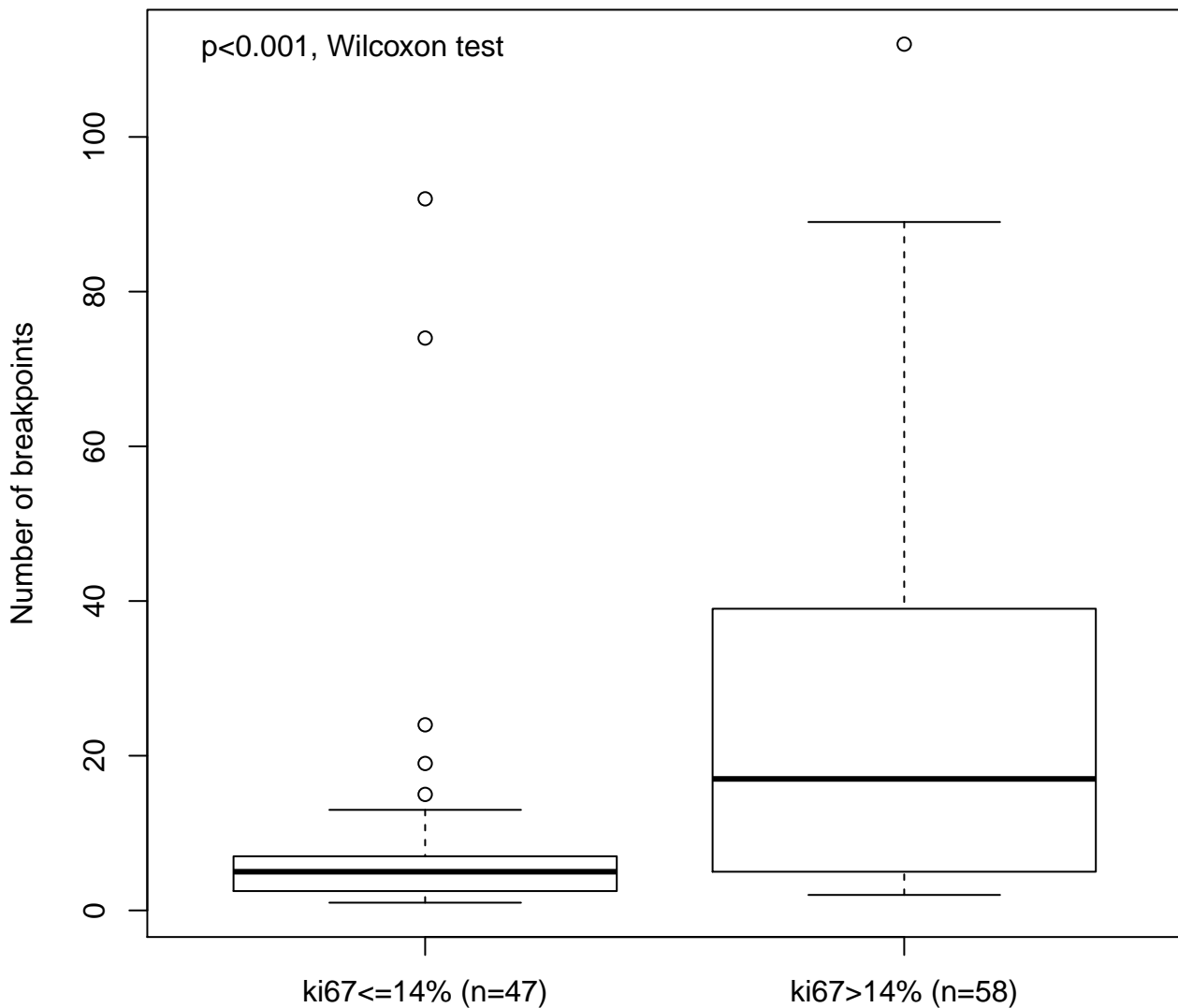

Supplement: Figure S1 — Number of breakpoints according to KI67. (PDF) [file pone.0076496.s003.pdf]

**Gene expression grade according to number of breakpoints**

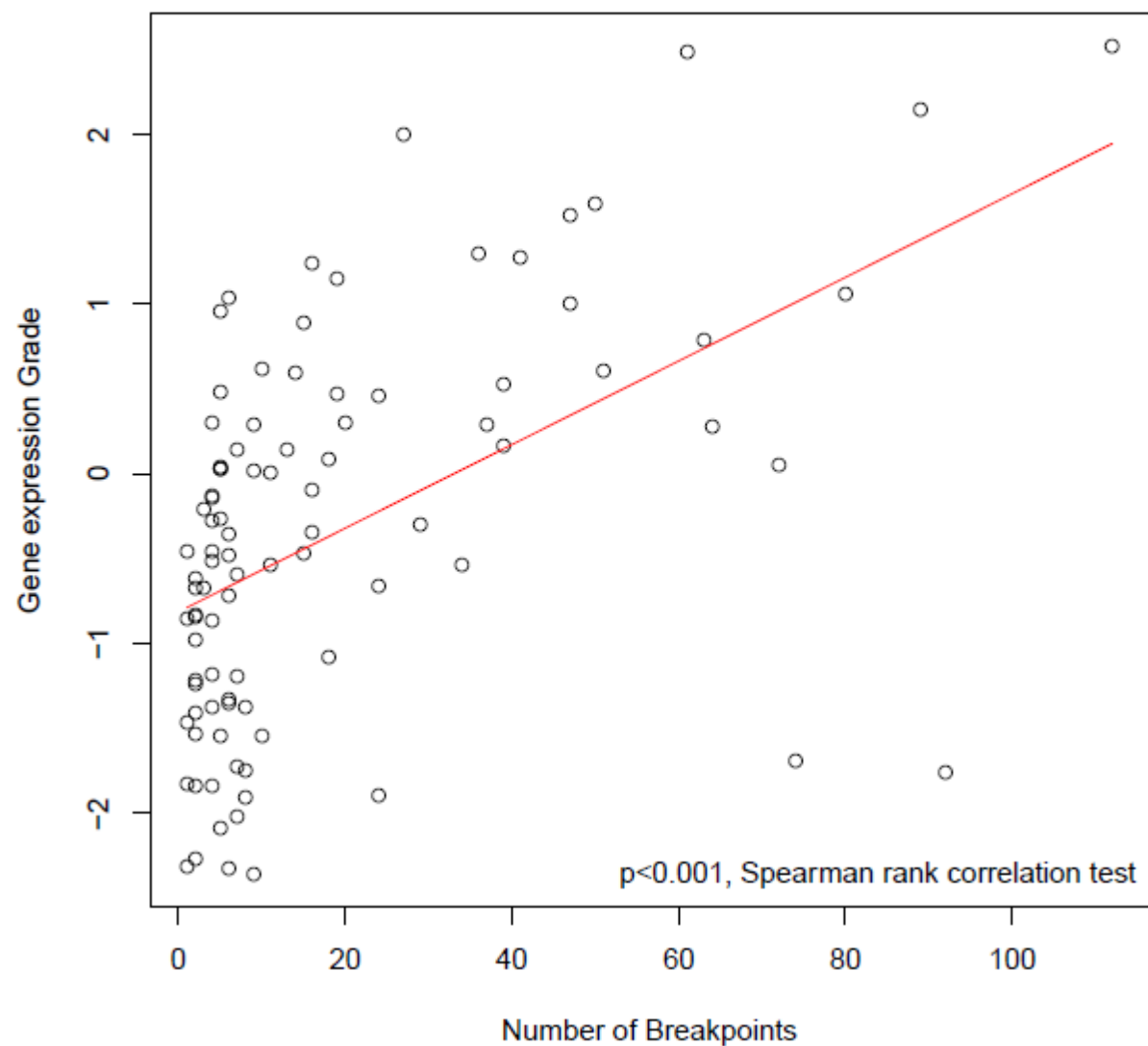

Supplement: Figure S2 — Gene expression grade according to number of breakpoints. (PDF) [file pone.0076496.s004.pdf]
